# Editorial: Urological cancer awareness month – 2022

**DOI:** 10.3389/fruro.2024.1278688

**Published:** 2024-05-20

**Authors:** Georgios-Ioannis Verras, Francesk Mulita

**Affiliations:** ^1^ General Surgery, Epsom and St. Helier University Hospitals, National Health Service (NHS) Trust, London, United Kingdom; ^2^ Department of Surgery, General University Hospital of Patras, Patras, Greece

**Keywords:** urological cancer, bladder cancer, renal cancer, next generating sequencing, prostate cancer

Urological cancer is currently one of the most frequently diagnosed forms of cancer worldwide and constitutes a significant burden of disease for public health. Bladder carcinoma, one of the most prominent types of urogenital cancers, is increasing in disease burden according to current epidemiological data, with a yearly spiking incidence that is expected to continue growing in the future, owing to demographic characteristics of the aging population, and continuous exposure to known risk factors (1). Prostate carcinoma, the most common of this category of cancers, is a particularly challenging form of cancer, with advanced-stage patients experiencing complex and debilitating forms of pain, in up to 90% of the cases (2). This pain’s very origins are also complicated in nature, with pain arising from the primary carcinoma itself, metastatic lesions, or the treatment efforts, with a small percentage of patients with chronic pain due to undiagnosed prostate cancer. The sheer numbers regarding the population-based epidemiological metrics and the individual-based specifics of urological cancer, as well as the excruciating pain syndromes described, constitute a significant burden of disease for our society, with increasing rates of diagnosis, and increasing years of life lost or lived with disability (3). These trends call for increased efforts in the early diagnosis and more effective management of urological carcinoma patients, be it from pre-operative chemotherapy, to palliative treatment adjuncts in advanced stages. The present Research Topic, celebrating the urological cancer awareness month, aims to bring into light novel treatments and diagnosis modalities for urogenital cancer, in the early forms of their development, to increase visibility of research efforts that might lead to earlier diagnosis, effective treatment, and ease of a great disease burden for our modern society. The editorial team is delighted to present some of the most exciting original works of research teams from around the world, focusing on urological cancer treatment and diagnosis.

Bladder urothelial carcinoma is the most commonly diagnosed form of bladder cancer in the world (4). Treatment efforts are mainly centered on trans-urethral resection of the primary tumor (whenever indicated), and postoperative chemotherapy or immunotherapy, with the use of the Bacillus Calmette-Guerin (BCG) adjunct. Still, recurrence rates for this type of cancer can reach up to 8,05% (5), with chemotherapy and maintenance regimens providing the most optimal survival results thus far. The original work by Moshnikova et al. describes a novel method of malignant cell detection and imaging of urothelial cell bladder carcinomas with promising implications as a therapeutic intervention as well. In their study, the authors utilize pH low insertion peptides (pHLIP) as a targeting system of the low-pH surface of cancer cells to detect urothelial bladder cancer cells *ex vivo*. Indocyanine green (ICG) paired with pHLIP allowed for near-infrared fluorescent imaging to take place, with a diagnostic accuracy of 98.3% of the specimens reviewed, providing an improvement in diagnosis rates of 17.3%, when compared to white light cystoscopy. No non-cancerous lesions were targeted by pHLIP-based imaging, while the differential diagnosis (ddx) list of diagnosed lesions included posttreatment effects of tissue necrosis, inflammation, and granulomatous tissue. The authors expanded their work by utilizing the RNA polymerase II inhibitor α-amanitin (6) as the delivered cytotoxic agent by pHLIP. They were particularly successful with cell lines having a 17p loss mutation, recording an increase in pH-dependent selective cytotoxicity of amanitin, indicating that their use of pHLIP can be expanded as a selective therapeutic intervention, with high sensitivity.

Magnetic resonance imaging is rising as the new standard in prostate cancer imaging and adjunct to definite diagnosis. Targeted biopsy techniques and biomarker adjuncts to MRI imaging (7) are rising in popularity, as transrectal ultrasound is proving to miss a clinically significant proportion of prostate tumors, as well as underestimates the aggressiveness of diagnosed tumors (8). Although better in diagnostic accuracy, MR-based modalities are often a logistic challenge, with few institutions being able to provide a streamlined service, largely due to involved costs. In their study, Sze et al. are looking into lower field strength MRI and its potential in targeted prostate biopsy. Their findings support that a portable MRI modality is equally as successful and useful in malignancy detection, at shorter operating times, without requiring a specialist urologist or radiologist present, thus lowering overall costs.

In a complex, quickly evolving and often challenging to diagnose cancer such as prostate cancer, personalized genome-directed treatments targeting oncogenic mutations can be an answer for patients requiring systematic therapies. Patients eligible for Poly (ADP-ribose) polymerase (PARP)-targeted treatments are those with specific germline or somatic gene mutations, detected by next-generation sequencing (NGS) (9). In the study of Griffin et al., the role of genome-directed treatments for prostate cancer is explored, specifically using the PARP inhibitors in a single-center study. Their work addresses one of the most controversial aspects of NGS diagnosis and genome-directed treatments, which is the degree that patient NGS will eventually lead to a patient receiving such treatment, as the proportion of variants that offer targeted therapeutic opportunities remains low. Genome-directed treatments are evolving rapidly, especially regarding the classification of a variant as actionable or not. Therefore, investigations for eligibility of treatment (in this case, NGS) must evolve accordingly to make a difference in the true number of patients receiving indicated GD treatment that can aid in determining disease progression and overall survival (10).

When studying a subtype of cancer that is as frequent and as diverse as bladder cancer, one must always assess that bladder cancer survivors are linked with a heterogeneous group of risk factors for carcinogenesis that contribute to multiple forms of cancer arising. In their work, Othmane et al. explore the epidemiological role of race and bladder cancer histology on the occurrence of a second primary cancer in bladder cancer survivors. Using 18 years of data from one of the biggest registries, they showed that Asian and Pacific Islander patients were more likely to develop a second primary cancer, with prostate cancer coming in first as the most frequent. The prevalence of the different types of malignancies also differed between races and between histological subtypes of bladder cancer. Epithelial histology was the most commonly associated subtype across all races. This finding can be explained by several factors, including the carcinogenic effects of chemotherapy and radiation treatment that is most often received by patients with epithelial bladder cancer (11–13). In the last few years, technological developments in the fields of surgery and urology have been rapid and continuously evolving. One of the most revolutionizing breakthroughs was the introduction of the IoT concept within the surgical and urological practice (14).

This Research Topic is a unique opportunity to focus on advancements in some of the most difficult-to-manage aspects of urological cancers; early identification, accurate diagnosis, efficient imaging, and novel treatments for advanced-stage carcinomas are all presented here.

## Author contributions

G-IV: Methodology, Writing – original draft, Writing – review & editing. FM: Conceptualization, Project administration, Writing – original draft, Writing – review & editing.
